# Promoting medical competencies through a didactic tutor qualification programme – a qualitative study based on the CanMEDS Physician Competency Framework

**DOI:** 10.1186/s12909-019-1636-5

**Published:** 2019-06-04

**Authors:** Angelika Homberg, Jan Hundertmark, Jürgen Krause, Merle Brunnée, Boris Neumann, Svetla Loukanova

**Affiliations:** 1 Department of General Practice and Health Services Research, University Hospital Heidelberg, Im Neuenheimer Feld 130.3, 69120 Heidelberg, Germany; 2 Abteilung Schlüsselkompetenzen und Hochschuldidaktik, Heidelberg University, Bergheimer Straße 20, 69115 Heidelberg, Germany

**Keywords:** Medical education, Peer teaching, Competencies, Tutor training, Procedural skills, Role competencies, CanMEDS

## Abstract

**Background:**

In peer-led tutorial courses, qualified medical students (“tutors”) provide their peers with opportunities to deepen their theoretical knowledge effectively and to practice clinical skills already in preclinical semesters. At the Medical Faculty of Heidelberg University, a structured medical didactic qualification programme prepares and trains future tutors for their responsibilities. This programme consists of four modules: 1. medical didactics and group leadership, 2. subject-specific training, 3. performance of tutorial courses as well as 4. collegial advice and reflection on the tutors’ activities. The aim of this study is to systematically analyse and present the development of role competencies for medical tutors based on the CanMEDS Physician Competency Framework through the didactic qualification programme.

**Methods:**

We applied a qualitative research approach to detect CanMEDS role competencies acquisition within the tutor qualification programme. The CanMEDS framework describes key competencies, grouped thematically under seven professional roles. Two tutors and three training coordinators independently assigned the individual modules of the tutor qualification programme to the key competencies of the CanMEDS framework. Tutors and training coordinators compared and discussed the allocations within the groups in a consensus finding process. All authors analysed the findings in order to find out the so-called “hidden curriculum”. The views of both groups are presented separately.

**Results:**

The training programme promotes the acquisition of competencies in all seven CanMEDS roles. The roles of the scholar and the leader are promoted in all modules. In addition, the first and fourth module focus predominately on the role of the collaborator, the second on the role of the medical expert and communicator, and the fourth on the role of the professional.

**Conclusions:**

The systematic analysis through assignment of the CanMEDS roles to the individual modules of the tutor qualification programme documents the comprehensive acquisition of competencies, not only with regard to the tutor activity, but generally with regard to the later role of the physician. The reflection on one’s own competency acquisition can support the promotion of corresponding competencies in the qualification programme and their transfer into the professional practice later.

## Background

During the first two years of medical education, tutorial courses give a gainful opportunity to students to deepen their acquired theoretical knowledge effectively and to practice clinical skills under the guidance of fellow students in their higher semesters [1–6]. Many publications show that student tutors are equal or even better in teaching certain medical skills then professional teachers, since they are closer to the learners and therefore better understand their learning requirements and needs [7–10]. In addition, tutors themselves can benefit from teaching, for example by developing professional attitudes and a better understanding of the theoretical knowledge acquired during their studies [11] and by repeatedly training practical skills [2, 12]. Furthermore, tutors benefit from the acquisition of didactic skills with regard to their future work as physicians and their professional career [13–15]. In principle, tutors are specifically prepared for their work as tutors through training, since the respective teaching quality in tutorial courses depends largely on the qualifications of the student tutors through suitable training programmes [16–18]. However, there is insufficient information in the literature on how the respective qualification of tutors is carried out and what content is taught [19, 20]. The questions also arises as to whether the increase in the tutors’ competencies is due to the tutors’ preparatory training or by the preparation, implementation and reflection of the tutorial courses themselves, and what is the contribution of the respective qualification modules.

In particular, there have been no studies to date that show which specific competencies, based on the CanMEDS role framework are promoted by the tutors’ training. The CanMEDS Physician Competency Framework offers a suitable analytical tool for this. It was developed in 2005 by the Royal College of Physicians and Surgeons of Canada as the basic framework for defining the role of the physician. It describes the fields of activity in the medical professional practice and the associated skills, based on seven roles [21]. Each role is represented by two to five key competencies, which describe the respective abilities, skills and attitudes. Since its introduction in 1996, the CanMEDS Physician Competency Framework has been adopted around the world and was introduced in Germany in the National Competency-Based Learning Objectives Catalogue for Medicine (NKLM) published for the first time in 2015. In the NKLM, the teaching content is systematically allocated to the respective CanMEDS role competencies in order to define which role competencies are acquired in the respective study sections. In this way, the allocation of competencies can be comprehensively presented over the course of the medical study and serves as a basis for further educational development.

In 2010, an integrated qualification programme for student tutors was introduced at the Heidelberg Medical Faculty and continuously refined and improved [22, 23]. On a total of seven training days with altogether 40 teaching hours, tutors are prepared for their teaching activities through training in didactics, group management, problem-oriented learning and physical examination techniques as well as in planning and performance tutorial courses and reflecting on their tutoring activities.

The aim of this paper is to systematically analyse and present the acquisition of competencies in the qualification programme modules for medical tutors at the Medical Faculty of Heidelberg University, based on the CanMEDS role framework, in order to show to what extent different medical roles are promoted in the respective modules.

The focus is on the following guiding questions:

1. Which CanMEDS roles are improved through the qualification programme for tutors?
2. Which module components create the largest share of competency development?
3. Are there different views concerning these topics between tutors and tutor training coordinators?

The answers can contribute…

1. for continuous improvement of the given curriculum,
2. for granting university credits for tutor-trainings,
3. to identify contents, which contributes to role-competency development,
4. to indicate differences between the official curriculum (views of the tutor training coordinators) and hidden curriculum (views of the tutors).

## Methods

### Tasks of the student tutors at the Heidelberg Medical Faculty

At Heidelberg Medical Faculty, student tutors from higher semesters are firmly integrated into the preclinical phase of the medical study, guiding small groups of 10 to 12 students for training of medical skills. Currently, tutors are trained to teach anatomy, sonography, practical skills as well as anamnesis and physical examination. The so-called Aal^Plus^ programme (living anatomy plus anamnesis) focuses on the teaching of physical examination techniques and clinical practical skills such as hand disinfection and blood sampling, exercising medical history with simulation patients and solving clinical cases on the basis of problem-oriented learning.

**Fig. 1:**
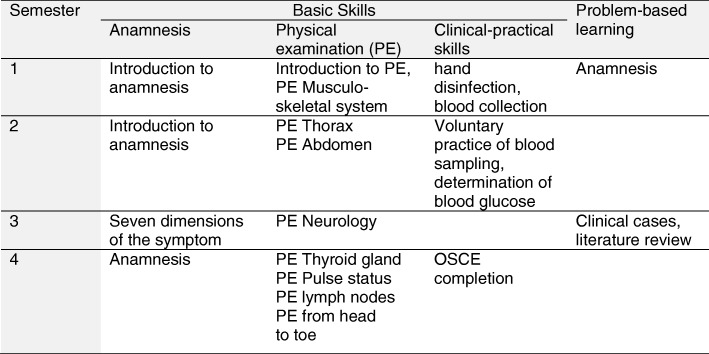
Overview on Aal^Plus^ tutorial course topics taught by the tutors

The aim of this Aal^Plus^ programme is to link the more theoretical content of the pre-clinical phase of the medical study with clinical and practical content in order to prepare the students for practical assignments from the third academic year onwards. The Aal^Plus^ tutorial courses are offered longitudinally over the first two years of study and are compulsory for every student (see Fig. 1). At the end of the second academic year, students have to pass an Objective Structured Clinical Examination (OSCE), which is completed with individual feedback to the respective students. The student tutors serve as examiners. The advantages of student tutors as examiners lie in their flexibility in terms of time and low costs, but require targeted preparation [16]. Overall, the tutors act independently and contribute substantial to the teaching of medical skills in the pre-clinical phase. This requires the appropriate personality and qualification of the tutors with regard to teaching and guiding groups as well as in technical knowledge in order to ensure the quality of teaching [24]. New tutors are selected through a complex recruitment process. Candidates have to be at least in the third study year. In the first step, a formal application, CV and motivation letter must be sent to the faculty staff. The documents are checked for completeness. In the second step, experienced tutors, who already know the candidates from the tutorial courses, evaluate the personality and suitability of the remaining candidates and divide them into following three groups: well suitable, conditionally suitable and less suitable. Depending on the need, the required number of new tutors were invited to the tutor qualification programme.

### Development and structure of the tutor qualification programme

Based on needs analyses, a programme consisting of four modules was developed and implemented since 2010 [22] in cooperation with the Department of Key Competencies and Qualification in Higher Education at the Heidelberg Medical Faculty. The programme currently consists of about 40 h of teaching time and contains basic didactic and subject-specific training as well as individual teaching activities of the tutors, including reflection units. Students’ participation in the basic didactic and subject-specific training is a requirement in order to be able to start working as tutor. These obligatory training courses are held in the beginning of each semester and are conducted by general practitioners, psychologists and educators for transferring an advanced level of clinical and didactic knowledge to the new tutors. Consultation and reflections on tutors’ activities are regularly offered by both, the Medical Faculty and the Department for Key Competencies and Higher Education in order to indorse the personal development and performance of the tutors. The tutors have the opportunity to evaluate the seminars and discuss problems and possible solutions at an annual final meeting. Tutors who complete the full qualification programme receive a Heidelberg Didactic Certificate for Tutors. The training concept has been continuously refined and improved since 2010 by a multidisciplinary team involving faculty staff and students.

### Detection of competency acquisition within the tutor training programme

A qualitative research approach [25] was applied to detect competency acquisition within the tutor qualification programme. Competencies themselves are based on estimates and assumptions; thus the presented data seems to be less hard facts than the result of a process of discussion and negotiation, involving all actors. It is therefore more important that the mapping process is described in a comprehensive way.

A four level-coding system, modified according to Kelley et al. [26, 27], was used to outline where and to which extend expected competencies are taught in the medical didactic qualification programme for medical tutors. It is based on the CanMEDS Physician Competency Framework for defining seven professional roles of the physician. Kelley’s coding system includes a further step: “Implementing the changes, create a plan to address the changes needed based on the analyses in step 4” [26] which is not applied yet, because organizational structures play a role in developing the implementation plan for any necessary or desirable curricular change. In addition, to carry out this step will not contribute to answer the guiding questions. In the following, the four steps of the curricular mapping process are described.

#### Planning

For continuous updating and further development of the tutors, the faculty members and training coordinators are responsible for the competency-oriented revision of the Aal^Plus^ tutor training programme. Faculty members and training coordinators decided to map the competencies gained in all components of the tutor qualification programme to receive a detailed overview on skills development. In order to capture both, the students’ and the teachers’ perspective, two tutors who have completed the entire training programme and three training coordinators who are predominantly responsible for at least one module were recruited for the mapping process and systematic analyses. All participants are familiar with the tutor qualification programme.

#### Creating the code

Each of the four modules of the qualification programme: medical didactics and group leadership, subject-specific training, performance of tutorial courses, and reflection on tutors’ activities, was subdivided into three to four thematic components (Table 1). The individual components as well as the key competencies listed in the CanMEDS Physician Competency Framework were compiled in a table. Each module component was assigned a column and each key competency a row. This table serves as a template for the allocation of competencies to the respective modules. The code for the map had been previously defined as follows:

- Although, allocation of the components of the modules to the competencies take place on the level of key competencies, the data shall be presented on the level of role competencies. Setting to a lower resolution of data representation ensures a higher level of reliability.
- To assign a module component to a role, at least one of the respective key competencies had to be allocated.
- Consensus had to be reached within but not between the groups.

**Table 1:** Assessment of professional role competencies acquisition in the tutor qualification programme’s module components

Module	Topic	Hours (+/−)	Components	Medical Expert	Communicator	Collaborator	Leader	Health Advocate	Scholar	Professional
1A	Medical didactics	5	Tasks and roles of a tutor	T	T	T, C	T		T	T, C
			Didactic basics of teaching and learning	T		T			T, C	
			Design of entry and exit phases of a tutorial course		T, C	T, C			C	
			Motivation of participants, experiences of experienced tutors	T	T, C	T, C	T		T, C	
1B	Group leader	5	Previous experience with groups and leadership, perception exercises		T, C	T	T		T, C	
			Rank dynamics, team roles and group phases		T, C	T, C	T, C		T, C	
			Group leadership and leadership styles	T	T, C	T, C	T, C		T, C	
			Dealing with resistance and disruptive factors	T	T, C	T, C	T, C	T	T, C	T
2	Subject-specific trainings	30	Planning and design of the topic-specific tutorial course	T	T, C	T	T, C		T, C	T, C
			Communication/transfer of the subject-specific contents	T, C	T, C	T	T, C	T	T, C	T
			Feedback, Integration of simulation patients	T, C	T, C	T, C	T, C	T	T, C	T, C
3	Performance of tutorial courses	20	Structured preparation for the respective tutorial courses in tandem	T	T, C	T, C	T, C		T, C	
			Preparation of materials and deepening of the content				T, C		T, C	
			Independent tutorials implementation in tandem	T	C	T, C	T, C	T	T, C	T
4	Reflexion	20	Consultation (Collegial advice)	T	C	T, C	T, C	T	T, C	C
			Collegial visits			T, C	T, C	T	T, C	T, C
			Final reflection		T	T	T	T	T, C	T
			Portfolios, self-reflexion und final report		T	T	T	T	T, C	T

*Note*. T: Respective module component promotes role competency according to the tutors’ assessment. C: Respective module component promotes role competency according to the training coordinators’ assessment

#### Data collection

In the first step, the two tutors (MB, JK) and three training coordinators (JH, AH, BN) independently allocated the respective components of the modules to the key competencies. In preparation for this, all participants received and studied the detailed CanMEDS role competency framework [21].

In a second step, each group of raters, the tutors (group T) and the training coordinators (group C), compared and discussed their results within their group since consensus was reached. Here, it was assessed which of the seven CanMEDS competency roles were promoted in the individual components of the modules. Each of the module components was assigned to a role if at least one key competency was implicitly or explicitly assigned in the respective module from the point of view of the tutors or training coordinators (Table 1), it means tutors and respectively training coordinators assume that this module promotes the particular role.

In the third step, the allocation of competencies done by the tutors and training coordinators was compared and discussed in order to find out the so-called “hidden curriculum”. The views of both groups are presented separately in Table 1.

#### Analyses of map

**Fig. 2:**
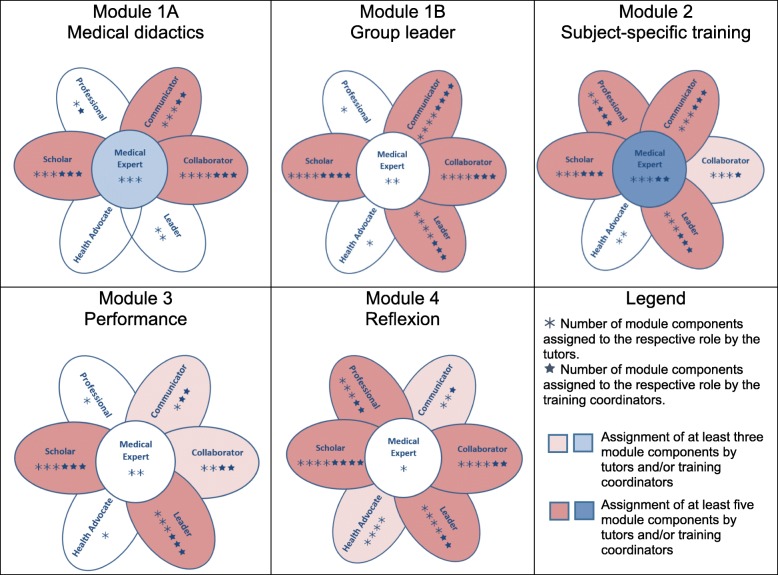
Promotion of CanMEDS role competencies in the individual training modules of the tutor qualification programme

All authors were involved in the analyses process and regard the data shown in Table 1 and Fig. 2 along the guiding questions described in the background section.

## Results

The tutor qualification programme promotes competencies of all seven CanMEDS roles. Overall, the comparison of the assessment of tutors and trainers showed a high level of agreement, although the tutors assigned more role competencies to the individual modules especially to module four. According to the assessment of both groups, the role of the scholar is promoted in all modules, the role of the leader from module 1B onwards. In addition, the first and fourth modules focus on the role of the collaborator and the fourth on that of the professional. In both groups, in module 2 is seen at most comprehensive acquisition of competencies, which is also reflected in the promotion of the role of the medical expert. The role of the health advocate is only assigned to individual modules by the tutors, but not by the training coordinators.

## Discussion

The CanMEDS Physician Competency Framework is proved to be an appropriate analytical tool for presenting the acquisition of competencies within the individual modules of the qualification programme, although its suitability for structuring teaching units is critically discussed [28].

Based on these results, the first question to be explored is which competencies are improved through the tutor qualification programme. With regard to all roles, it is not surprising that the roles of the scholar and the leader are in focus in all modules, both from the perspective of the students and the training coordinators, with the exception of the leader in module 1A (medical didactics). The training content explicitly addresses teaching and leadership competencies that can be tested and deepened in the performance of the tutorial courses. However, McConnell et al. surveyed medical and surgical specialists about their learning needs to find out intrinsic CanMEDS competencies. Most of the perceived learning need responses were coded by qualitative content analyses to the scholar and leader role. The participants described needs relating to providing feedback, developing teaching skills as well as leadership skills and practice management [29]. This emphasises the key importance of these two roles for the later career. Furthermore, as far as the view of the tutors is concerned, all roles are supported during the four modules, even though with different focus. This shows that tutors should be encouraged to complete the full training programme to enable extended competency acquisition.

With regard to the second guiding question, in the second module (subject-specific training) many module components were assigned to the individual roles. This is possibly related to the fact that the subject-specific training module simultaneously imparts didactic skills and deepens medical skills. Thus, tutors learn to react adequately to different challenges and train the students how to enter into medical roles themselves. Ochsmann et al. examine the connection between self-assessed deficits in medical skills and knowledge, and the feeling of preparedness of junior doctors. About 60% of the doctors felt poorly prepared for post graduate training. This makes the transition from studies to professional practice considerably more difficult [30]. In particular, the tutor qualification programme provides a various spectrum of individual learning situations like specific feedback situations and learning how to deal with simulation patients. This explains why the role of the medical expert is most clearly promoted in this module. This role plays a central role in the CanMEDS framework, as it integrates all other roles and includes both, action competencies and corresponding professional values (literally from the framework (21 p9): “As medical experts, physicians integrate all of the CanMEDS roles, applying medical knowledge, clinical skills, and professional attitudes in their provision of high-quality and safe patient-centered care. Medical expert is the central physician role in the CanMEDS framework and defines the physician’s clinical scope of practice”). Tutors learn by teaching and not only consolidate what they have learned through their studies but also get experts due to the different requirements in the tutorial courses [19].

Comparing the views between tutors and tutor trainers, the role of health advocate is assigned less frequently by the trainers than by the tutors. This is probably due to the different perspective of the student tutors, since they associate their tutoring activities with the medical activity in a different way through observations than external trainers. In class with student tutors, they see themselves in the role of mediators, answer questions, respond to the students, promote understanding and convey facts - just like the doctor does to his patients in a clinical setting [31].

The roles of medical expert and professional were also assessed differently by the tutors and training coordinators. From the perspective of the tutors, these two roles are addressed in all module components. This could be based on the self-assessment of the tutors, who actively deal with these roles themselves and anticipate the tutorial courses implementation already in the first module. From the tutors’ perspective, the specific content-related trainings impart expert knowledge and professionalism, which is reactivated before and during each tutorial course. In comparison, the training coordinators see the competency of the medical expert only in connection with the tutorial courses implementation and that of the professional, related to the reflection and thus the reflection and discussion of what has been learned through consulting and observation. Tutors estimate their competency acquisition as high, especially in the fourth module, where tutors reflect their activities, are widely differing views. We assume that tutors learn much more through collegial advice and self-reflection then the tutor trainers expect [32].

Overall, it was shown that the structured training programme for tutors makes a considerable contribution to their qualification for later medical work. Doctors working in hospitals particularly emphasised the importance of the roles of medical experts for practical work [33]. From this it can be concluded that tutors are particularly well prepared for the upcoming practical year and the later medical activity.

The role of the scholar can also be transferred from tutoring to medical work, since parallels can be found between the social roles of the students and patients or those of the tutors and doctors [14, 19]. Both, tutors and doctors are obliged to convey knowledge in an understandable, collaborative way, actively involving the students or patients in order to reach a consensus. Both sides are responsible for the successful interaction process and use similar communication strategies, such as indirect communication. Furthermore, the preparations for a tutorial course by acquiring knowledge and dealing with the contents to be included and the integration of simulation patients as well as the actual execution of the tutorial course are comparable to the preparations for doctor-patient consultations, since in both cases is required handling of the unpredictable and a flexible reaction. It is also conceivable to transfer the acquired competencies to the leadership and direction of a practice team as well as the foresighted handling of time- and other material resources.

### Limitations

Since qualitative analyses are interpretative by nature, it is possible that different analyses considerations yield different results. However, we captured and discussed the perspective of tutors and training coordinators, so that a total of five persons were involved in the analyses process. In addition, the data categorization procedures were rule- and theory guided.

## Conclusion

The explicit assignment of the CanMEDS roles to the individual module components underlines the comprehensive competencies acquisition by the tutors with regard to the later medical role through the medical didactic qualification programme. The structured qualification programme contributes considerably to this. As trainers, the tutors themselves serve as “models” for the implementation of the CanMEDS roles. By reflecting on one’s own competency acquisition, can be supported the promotion of corresponding competencies in the qualification programme and the transfer into the professional practice.

## Data Availability

The datasets used and/or analysed during the current study are available from the corresponding author on reasonable request.

## Abbreviations

Aal^Plus^: Anatomie am Lebenden (living anatomy) plus Anamnese
NKLM: National Competency-Based Learning Objectives Catalogue for Medicine
OSCE: Objective Structured Clinical Examination
PE: Physical Examination
